# Onset Dynamics of Action Potentials in Rat Neocortical Neurons and Identified Snail Neurons: Quantification of the Difference

**DOI:** 10.1371/journal.pone.0001962

**Published:** 2008-04-09

**Authors:** Maxim Volgushev, Aleksey Malyshev, Pavel Balaban, Marina Chistiakova, Stanislav Volgushev, Fred Wolf

**Affiliations:** 1 Department of Phychology, University of Connecticut, Storrs, Connecticut, United States of America; 2 Department of Neurophysiology, Faculty of Medicine, Ruhr-University Bochum, Bochum, Germany; 3 Faculty of Mathematics III, Ruhr-University Bochum, Bochum, Germany; 4 Institute of Higher Nervous Activity and Neurophysiology Russian Academy of Sciences, Moscow, Russia; 5 MPI for Dynamics and Self-Organization, Faculty of Physics, University of Göttingen, Bernstein Center for Computational Neuroscience, Göttingen, Germany; Vrije Universiteit Amsterdam, Netherlands

## Abstract

The generation of action potentials (APs) is a key process in the operation of nerve cells and the communication between neurons. Action potentials in mammalian central neurons are characterized by an exceptionally fast onset dynamics, which differs from the typically slow and gradual onset dynamics seen in identified snail neurons. Here we describe a novel method of analysis which provides a quantitative measure of the onset dynamics of action potentials. This method captures the difference between the fast, step-like onset of APs in rat neocortical neurons and the gradual, exponential-like AP onset in identified snail neurons. The quantitative measure of the AP onset dynamics, provided by the method, allows us to perform quantitative analyses of factors influencing the dynamics.

## Introduction

The generation of action potentials (APs) is a key process in operation of nerve cells and communication between neurons. Our contemporary understanding of the AP initiation is based on the Hodgkin-Huxley theory [1]–[3]. An AP is initiated when a neuronal membrane is depolarised above the threshold for activation and opening of voltage-gated sodium channels. This supra-threshold depolarisation starts an avalanche-like process: opening of sodium channels leads to further depolarisation of the membrane and thus to opening of more channels. Although the original formulation of the Hodgkin-Huxley theory was based on the data from squid axons, the same formalism has been adopted for APs in all vertebrate nerve cells, including neocortical neurons in mammals [e.g. 4]–[6]. Recent findings indicate that action potentials in central mammalian neurons have a much faster onset dynamics than the typically found in models of Hodgkin-Huxley type [7], [8]. Here we describe a novel method which quantitatively differentiates the onset dynamics of action potentials. This method captures the difference between AP onset dynamics in rat neocortical neurons and in identified snail neurons: fast, step-like onset of APs in rats, but slow, exponential-like onset in snails. Quantification of the AP onset dynamics, provided by the method, will allow to study the contribution of factors which underlie the dramatic difference between AP initiation in rat and snail neurons, and to gain better understanding of intimate mechanisms of AP initiation in neocortical neurons.

## Results

**Figure 1:**
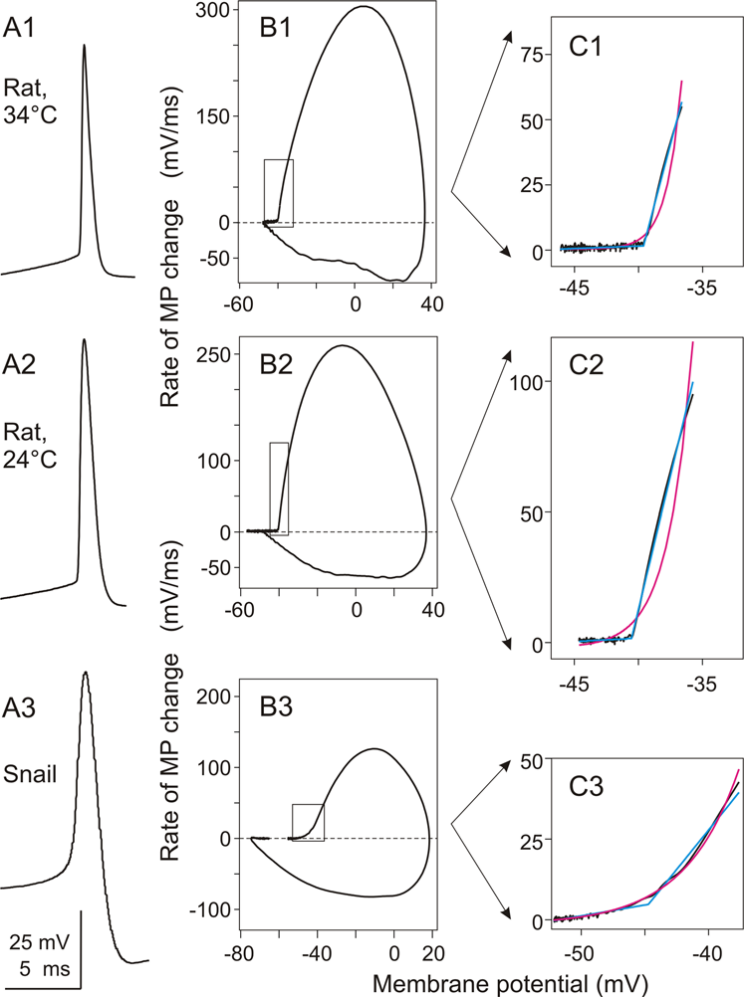
Action potentials recorded in neurons in rat neocortex (A1–C1 and A2–C2) and in snail neuron (A3–C3) have different onset dynamics. A: Waveform of the action potentials recorded in two rat neocortical neurons at 34°C (A1) or 24°C (A2) and in a small cerebral neuron (20 µm soma diameter) in snail (A3). B: Phase plot representation of the APs from A. C: Close-up of the initial portion of the phase plots indicated with boxes in B. Magenta lines show exponential fits, Cyan lines piecewise linear fits to the data. Ratio of errors of exponential fit over the piecewise-linear fit were 8.22 in C1, 12.3 in C2 and 0.55 in C3. Note different onset dynamics of the APs: rapid, step-like in rat neurons, but slow, exponential-like in the snail neuron.

Action potentials in mammalian central neurons are characterized by an exceptionally fast onset dynamics [7], [8]. Figure 1, A1,A2 show APs recorded in typical pyramidal neurons in slices of rat neocortex at close to physiological temperature (34°C, Fig. 1 A1) and at room temperature (24°C, Fig. 1, A2). The fast, step-like nature of neocortical AP initiation stands out especially clear in the phase-plot representation, in which the rate of change of the membrane potential is graphed against the instantaneous value of the membrane potential (Figure 1, B1, B2). This type of AP onset dynamics was observed in both regular spiking an fast spiking neocortical neurons (data not shown). In marked contrast to the step-like onset of AP in neocortical neurons, the onset of AP in a snail neuron is slow, with a gradual build up of potential at the AP beginning (Figure 1, A3). The difference between rat neocortical cells and snail neuron in the onset of AP is most clearly seen in the phase-plots (Figure 1, compare B1, B2 to B3). To formalise this difference in the AP onset dynamics, we took a phenomenological approach, and fitted the initial portion of the APs in phase-plot representation with two functions, an exponential and a piecewise linear. These two functions have minimal number of parameters and describe two fundamentally different processes. . Figure 1, C1–C3 show the results of these fits to the APs in rat and snail neurons. The rapid, step-like onset of AP in rat neocortical neurons is fitted well by the piecewise linear function, but the exponential fit is very poor. Fitted piecewise linear function coincides with the data almost completely (cyan and black lines in Fig. 1 C1 and C2), while exponential function essentially fails to describe the AP onset dynamics in rat neocortical neuron (magenta line in Fig. 1 C1 and C2). Fitting the onset of a snail neuron AP gives a very different picture (Figure 1, C3). For the snail AP, an exponential function provides a very precise fit to the data (magenta and black lines in Fig. 1, C3), but the fit with a piecewise linear function is of inferior quality (cyan in Fig. 1 C3). The above examples were typical for our sample: the onset of APs in rat neocortical neurons was fitted better with the piecewise linear function, while the AP onset in snail neurons was better fitted with the exponential function. As a quantitative measure of this difference in AP onset dynamics, we used the ratio of the error of the exponential fit to the error of the piecewise linear fit. This ratio is high when the AP onset dynamics is step-like and the piecewise linear fit is better (e.g. 8.22 and 12.3 in the examples shown in Fig. 1, C1, C2), but low when the AP onset is smooth, and the exponential fit is good (0.55 in the example in Fig. 1, C3).

This difference in the AP onset dynamics between rat and snail neurons cannot be explained by the difference in recording temperature. APs in rat neocortical neurons, recorded in temperature range between 20°C and 35°C invariably exhibited step-like onset dynamics (e.g. Fig. 1). The APs in identified snail neurons, recorded at temperatures of the overlapping range (20°–25°C) typically had slow onset dynamics (Fig. 1; see below for further examples). Moreover, the difference in the AP onset dynamics between rat neocortical neurons and snail cells cannot be attributed solely to the difference in soma size. In snail, slow AP onset dynamics was characteristic not only for large cells (soma >50 µm), but also for small neurons, comparable in size to the somata of rat neurons (e.g. Fig. 1 A3–C3, a neuron from cerebral ganglion with 20 µm soma).

**Figure 2:**
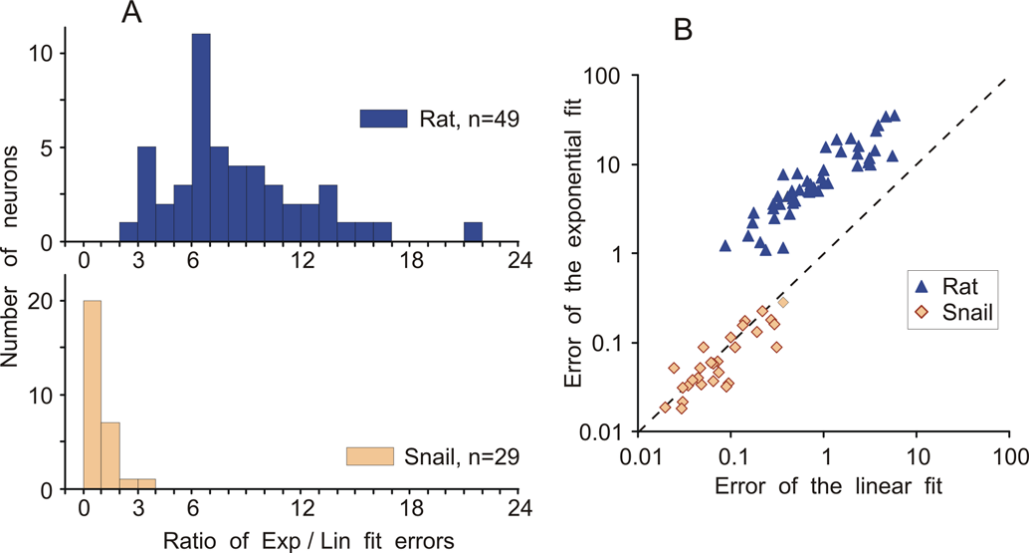
Population analysis: Ratio of the errors of exponential fit over the piecewise-linear fit for rat and snail neurons. A: Distributions of the ratio of errors of the exponential fit and piecewise-linear fit of the initial part of APs for 49 rat neocortical neurons and 29 snail neurons. B: Scatter plot of the error of exponential fit against the error of the piecewise linear fit for the neurons from A. Notice double-logarithmic scale. Each point represents data for one neuron. Colour code is the same in A,B.

Population analysis revealed a clear and consistent difference in the quantitative measure of the AP onset dynamics between rat and snail neurons. For 49 rat neocortical neurons, the averaged ratio of exponential to linear fit errors was 8.46±3.87 (mean±SD, n = 49), and for 29 snail neurons, the averaged ratio was 0.96±0.57 (n = 29). This difference is highly significant (p<0.0001, Kolmogorov-Smirnov Z = 4.121; ANOVA: F = 55.3). Moreover, the distributions of the ratio values for the rat and the snail neurons (Figure 2A) have very little overlap: for all but one rat neuron the ratio of exponential to linear fit errors was higher than 3 (the only value below 3 was 2.26) while for all but two snail neurons the ratio was below 2 (two values above 2 were: 2.1 and 3.15). The difference between rat and snail neurons in the onset dynamics of APs stands out very clearly in the scatter plot in Figure 2B, where the errors of exponential and linear fits for each neuron are plotted one against the other. In the scatter plot, the clouds of points representing rat and snail APs form two different, non-overlapping clusters. These results demonstrate that there are consistent differences in the AP onset dynamics between rat neocortical neurons and identified neurons of snail. The ratio of the errors of exponential and piecewise linear fits captured this difference and provided a quantitative measure for the AP onset dynamics.

**Figure 3:**
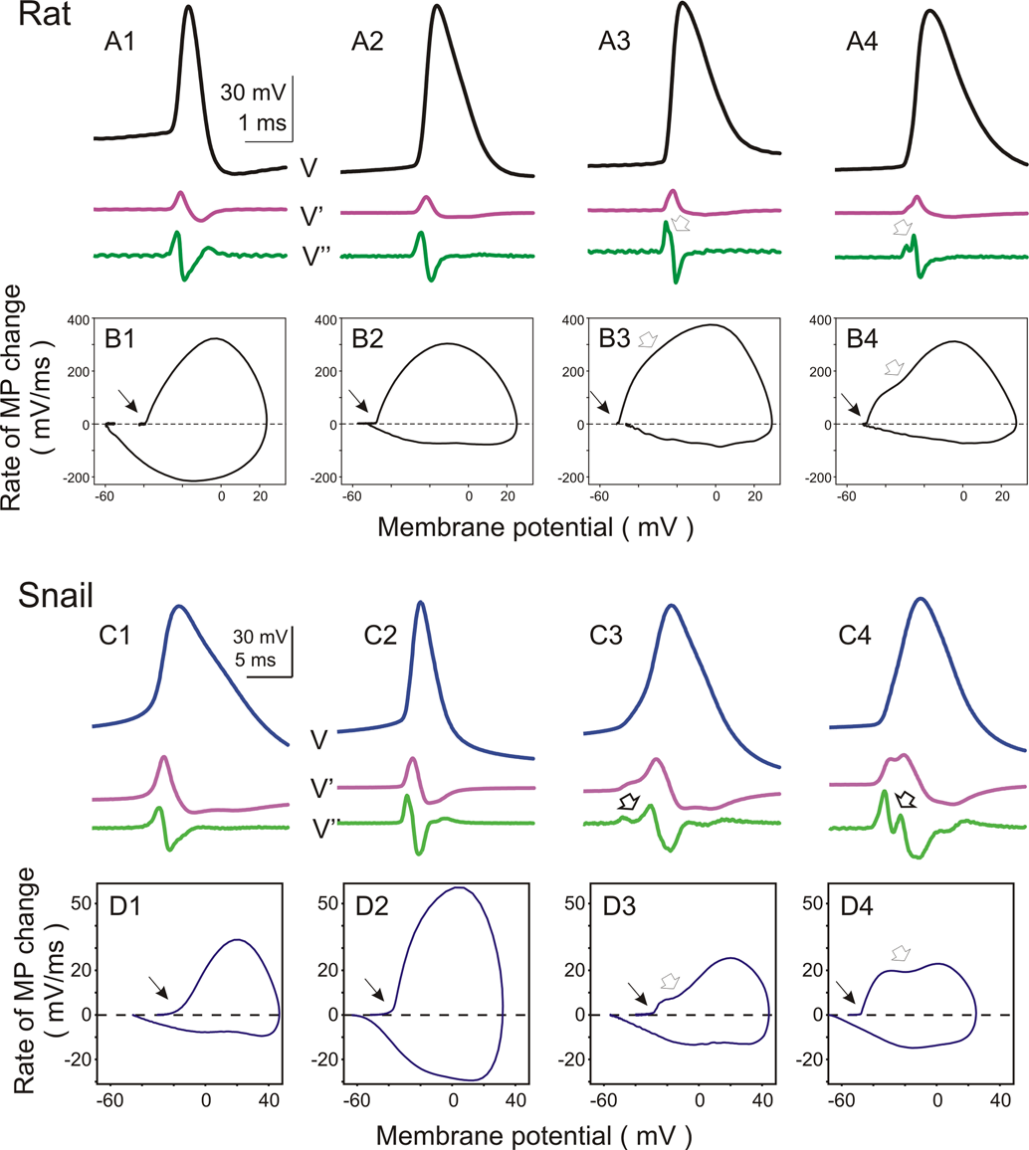
Action potentials in either rat and snail neurons have different waveforms. A, B: Action potentials recorded in 4 different neurons from rat neocortex. In A, voltage traces (V) are shown together with their first (V') and second (V”) derivatives. In B, phase plots of action potentials from A are shown. Ratio of errors of exponential fit over the piecewise-linear fit for these neurons were: 7.27; 6.07; 6.95; 3.46. C, D: Action potentials recorded in 4 identified snail neurons and their phase plots. Recordings from visceral ganglion neuron Visc5 (C1,D1); Left pedal ganglion neuron LPd2 (C2,D2); Right cerebral serotonergic neuron RC5HT (C3,D3) and Left pedal ganglion neuron LPd7 (C4,D4). Notice 8-fold difference in the Y-scale between B and D. In A–D open arrows indicate double-peak on the second derivative trace and bimodal shape of the phase plots. For both rat and snail neurons, the APs are sorted from left to the right by the increasing strength of manifestation of the double-peak on the second derivative trace and bimodal shape of the phase plots (open arrows). Ratio of errors of exponential fit over the piecewise-linear fit for 1–4 were: 0.79; 0.70; 0.92; 3.14. Notice that all APs in rat neocortex neurons have a sharp onset, irrespective of the presence and expression of the double peak in the phase plots.

In both rat and snail neurons, the APs recorded at the soma expressed different waveforms (Figure 3). The upstroke of APs may exhibit a non-monotonous voltage increase, suggesting a contribution of at least two distinct processes to the AP rising phase. These two components manifest themselves as double-peaks in the second derivative trace (Figure 3, A3,A4 and C3,C4, open arrows) and two humps in the phase-plot (Figure 3, B3,B4 and D3,D4, open arrows). A double-component rising phase is characteristic for antidromically evoked APs, and is attributed to the fact that APs may be generated not at the soma, but in the axon initial segment or the first node of Ranvier in mammalian neurons [e.g. 9]–[18] or down the neurite in snail neurons [e.g. 19]–[22]. In this scenario, the “initial segment” spike is generated first, and antidromically invades the soma before the “somato-dendritic” spike is generated [9], thus leading to a double-component shape of the AP upstroke. However, in many rat and snail neurons APs did not exhibit clear signs of double-component initiation and their derivatives changed monotonously during the upstroke (Fig. 3, A1, C1). In this respect, the recorded APs covered the whole range of the shapes of the second derivative changes, from a single-peak (Fig. 3, A1, C1), through intermediate cases (Fig. 3, A2, C2), to a double-peak (Fig. 3, A3,A4 and C3,C4). Interestingly, the presence of the double-peak second derivative had differential relation with the AP onset dynamics in rat as compared to snail neurons. In rat neurons the presence of the double-peak second derivative was not correlated with AP onset dynamics. Despite the variability in the details of their rising slopes, all APs in all neocortical neurons expressed a sharp, step-like onset dynamics (Figure 3, B1–B4). To address this issue quantitatively, we have compared the ratio of the errors of exponential and piecewise linear fits in 15 neocortical neurons with clearly single-component and 10 neurons with clearly double-component APs. This comparison did not reveal any significant difference between the onset dynamics of single-component and double-component APs (8.57±3.97 vs. 7.78±4.09, p>0.1). In snail neurons, however, the presence of a double-peak second derivative was correlated with the AP onset dynamics. The monotonously rising APs expressed mostly smooth onset dynamics, while the APs with pronounced double-peak second derivative had faster onsets (Figure 3, D1–D4). Two snail APs with the ratio of the errors of exponential and piecewise linear fits above 2, had clearly bi-modal second derivative (e.g. Fig. 3, C4,D4). We note, however, that onset dynamics of even the fastest snail APs was significantly more gradual than the onsets of typical neocortical APs (see Figure 2).

## Discussion

We describe a novel method of analysis which quantitatively differentiates the onset dynamics of neuronal action potentials. This method (i) captures the difference between the fast, step-like onset of APs in rat neocortical neurons and slow, exponential-like AP onset in identified snail neurons, and (ii) provides a quantitative measure of the AP onset dynamics, thus allowing for quantitative analysis of factors influencing the dynamics of AP initiation in nerve cells.

### Snail neurons as reference

To establish a measure of AP onset dynamics, we have used as a “reference” APs recorded in identified neurons in a snail *Helix lucorum*, and compared AP onsets in rat neocortical neurons and in snail neurons. A number of reasons speak in support of this choice. First, large invertebrate neurons represent a classical object for studying AP generation, and canonical Hodgkin-Huxley theory has been developed for the membranes of invertebrate neurons [3]. APs in these neurons typically express slow, canonical onset dynamics. Analysis of snail and rat neurons allows to cover a broad range of different AP onset dynamics. Second, the large size of snail neurons allows double- and triple- electrode recordings from the neurite and soma of one neuron [e.g. 19], [20], making the AP initiation and propagation experimentally accessible. Moreover, in many snail neurons the APs are initiated not in the soma, but in the neurite, and backpropagate to the soma [e.g 19]–[22], making them in that respect similar to the neocortical neurons [e.g. 9]–[15], [17], [18], [23]. In combination with the possibility of recording from neurites, this preparation allows direct electrophysiological investigation of the consequences of distal AP initiation and invasion on the somatically recorded AP waveforms (Malyshev et al., ms in preparation). One further argument for using snail neurons as a reference is that in living organisms even the APs with most different properties are compatible with normal neuronal functioning, as assured by the “control” of evolution. This balanced, evolutionary-controlled variation of parameters which are responsible for generation of APs is advantageous as compared to a possibility of systematic modification of the parameters in computer simulations. For these reasons, snail neurons represent a useful reference, to which the AP initiation in neocortical neurons can be compared.

### Exponential vs. piecewise linear fits as a measure of AP initiation dynamics

We have used the ratio of errors of exponential to piecewise linear fits of the initial portion of the AP in phase plot representation as a measure of the AP initiation dynamics. The choice of these two functions, although empirical on the first place, is supported by several reasons. First, both exponential and piecewise linear functions have minimal number of free parameters (actually in each case only one parameter was fitted, see Methods). Second, they describe fundamentally different processes, an avalanche-like process with smooth onset dynamics, and a step-like process with threshold-like onset dynamics. In fact, a slow AP onset, with an avalanche-like dynamics is a genuine property of a broad range of models with Hodgkin-Huxley-type activation kinetics of voltage-gated Na+ channels [3], [24], [25]. In contrast, recent experimental data show that the AP initiation dynamics in central neurons of mammals appears step-like, and faster than the canonical activation kinetics of voltage-gated Na+ channels can explain [7], [8]. Third, experimental data were always approximated well by at least one of these functions. Moreover, the ratio of errors of the two fits allowed us to quantify the difference of the AP onset dynamics in neocortical neurons as compared to snail neurons. This difference is obvious by visual inspection of the phase plots, but the ratio of errors of the two fits gives a quantitative measure and a formal criterion for segregation of neurons in two groups according to the type of the onset dynamics of their APs.

### Onset dynamics of APs with different waveforms

In vertebrate central neurons, APs are typically initiated in the proximal part of the axon not far from the soma: 35 µm from the hillock in layer 5 pyramidal cells in rat neocortex [18]; 35–50 µm from the soma in layer 5 pyramidal cells in ferret prefrontal cortex [23]; 75 µm from the soma in CA3 pyramids in rat hippocampus [16]; 75 µm from the soma, in the first node of Ranvier, in cerebellar Purkinje cells in rats [15], or closer to the soma, in the axon initial segment in Purkinje cells in mice [17]. Also in snail neurons, the AP initiation site may be located down the neurite [e.g. 19]–[22]. Propagation of distally initiated APs back to the soma may lead to the double-component shape and double-peaked second derivative of the AP rising phase. In theory, an interplay of activation/inactivation of sodium and fast A-type potassium channels, or activation of sub-populations of sodium [e.g. 14] or calcium channels could also contribute to the double-component shape of the AP rising phase. The first possibility is supported by the long-known presence of double peaks in the second derivative trace of antidromically evoked APs [e.g. 9]–[11]. In our sample, APs from both rat and snail neurons covered the whole range of wave shapes – from monotonous to well expressed double-component rising slopes. The expression of the double-peak in the second derivative was related to the AP onset dynamics only in snail, but not in rat neurons. In the snail neurons, monotonously rising APs had always smooth onset, while APs with double-component upstroke had faster onset dynamics. This suggests, that in the snail neurons, the main factor determining the onset dynamics of somatic APs is the location of AP initiation site (Malysev et al, ms in preparation). In rat neurons, however, in which APs of any waveform invariably had sharp, step-like onset, contribution of the distal AP initiation to the onset dynamics of somatic AP is less clear. Recent studies of length constants of axons of pyramidal neurons in the neocortex [23] and granule cells in the dentate gyrus [26] report values >400 µm. This means, that AP initiation site in neocortical neurons is located only about 1/10 of the length constant away from the soma. Although resistance of the cell membrane and cable properties of the axon change dramatically during the build-up of the depolarisation just before the generation of somatic AP, the rising phase of an AP initiated about 35–50 µm away from the soma might be directly “seen” in the somatically recorded signal. Thus, the onset dynamics of AP in the soma of rat neocortical neurons may reflect a combination of at least 3 different processes taking place before the peak of somatic AP is reached: direct reflection of membrane potential changes at the initiation site, back-propagation of the AP from the initiation site and generation of the somatic AP. The relative contributions of these factors may vary from one cell to another, and remains to be determined.

In conclusion, we demonstrated here a novel method that provides a quantitative measure of the AP onset dynamics, and thus allows to quantitatively study the contribution of the above as well as other possible factors which influence the dynamics of AP initiation in nerve cells.

## Materials and Methods

All experimental procedures used in this study were in accordance with the guidelines published in the European Communities Council Directive (86/609/EEC, 1986) and were approved by a local animal welfare committee (Bezirksregierung Arnsberg, Germany).

### In Vitro rat slice experiments

Recordings were made in slices of rat neocortex, containing the visual cortex. The details of slice preparation are described elsewhere [27], [28]. The perfusion medium contained (in mM) 125 NaCl, 2.5 KCl, 2 CaCl_2_, 1.5 MgCl_2_, 1.25 NaH_2_PO_4_, 25 NaHCO_3_, 25 D-glucose and 0.5 L-glutamine, and was aerated with 95% O_2_ and 5% CO_2_ bubbles. Recordings were made at 28–35°C or at room temperature (20–24°C). Patch-electrodes were filled with a solution containing (in mM) 127 K-Gluconate, 20 KCl, 2 MgCl_2_, 2 Na_2_ATP, 10 HEPES, 0.1 EGTA and had a resistance of 3–7 MΩ. Whole-cell recordings were made with patch electrodes under visual control. Action potentials were evoked by injection of intracellular pulses or by synaptic stimulation. For synaptic stimulation, bipolar tungsten electrodes were positioned below or aside the recorded cell. Cells of different layer location and morphology were visually pre-selected for recording using visual control using Nomarski optics and infrared videomicroscopy [29]. The intracellular signals were recorded with (Axoclamp 2B, Axon Instruments, USA, and additional DC-amplifier, total gain ×20 to ×100), low-pass filtered at 3–10 kHz and digitized at 20–100 kHz (DigiData 1200 and PClamp 6, or DigiData 1322A and PClamp 10, Axon Instruments) to store in a computer.

### Snail experiments

The experiments were performed on the mature specimens of *Helix lucorum* L. weighting 30–35 g. Snails were originally collected in Crimea and maintained in an active state in the Institutes' animal facility. Before dissection animals were anaesthetized by injection of isotonic MgCl_2_ (∼15% of the animal weight). The central ganglionic ring was removed from the animal and pinned onto a silicone-elastomer (Sylgard)-coated dish. Connective tissue sheath was partially removed using fine forceps and scissors. To facilitate further desheathing, the ganglia were treated with Protease (0.25 mg/ml; Type XIV, Sigma) for 10 min at room temperature and washed out, and then the fine sheath was completely removed. The CNS was bathed in a saline solution containing (in mM) 100 NaCl, 4 KCl, 7 CaCl_2_, 5 MgCl_2_, and 10 Tris-HCl buffer (pH 7.6). Electrophysiological recordings started at least 60 min after the dissection. Intracellular recordings were made at room temperature (20–25°C), using standard electrophysiological techniques. Neurons were impaled with glass microelectrodes filled with 2 M potassium acetate (tip resistance, 15–25 MOhm). Intracellular signals were recorded with several different preamplifiers (Neuroprobe 1600, A-M Systems; Bramp 01R, NPI or SEC 05LX, NPI), digitized at 10–20 kHz, and stored on computer (Digidata 1320A A/D converter and Axoscope 8.0 software, both from Axon Instruments, USA). Identified neurons were numbered in accordance with the published maps [30].

### Data analysis

For the analysis we used single or averaged APs from the recordings with stable membrane potential and stable AP threshold and waveform. If recorded at <100 kHz sampling rate, the data were interpolated to a resolution of *dt* = 10 µs using the MatLab spline interpolation function. For each AP we computed the temporal derivative *dV/dt*:
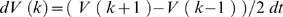



Plotting the *dV/dt* against the instantaneous membrane potential value (*V*), yielded a “phase plot” representation of an AP (e.g. Figures 1B, 3B, 3D). The initial portion of the AP in the phase-plot representation was fitted with an exponential and a piecewise linear function. Selection of the AP portion for the fit has been made in three steps. First, we identified the AP peak and maximal dV/dt. Second, we defined the AP onset, by fitting the voltage trace from −5 ms to −0.1 ms back from the AP peak with a continuous piecewise-linear function (see below). The breaking point of that fit was taken as the time point of AP onset, and membrane potential value at this point as the AP voltage threshold. This formal procedure identified the “kink” – when present – at the AP beginning. For the APs with smooth onset, in which case any single AP lacks a formal threshold, the onset found by the procedure corresponded well to the expert estimation. Third, using that formally identified AP onset, we selected a portion of the voltage trace starting −5 ms (or −10 ms for some very broad snail APs) before the onset, to either the point at which dV/dt reached 20–30% of the maximal value, or the voltage changed by 3–10 mV above the threshold. Special attention had been paid to set this latter limit before the rightwards curving of the AP in the phase plot. The ratio of errors of the exponential to the piecewise linear fit (see below) was little affected by small variations of the setting of that range. However, for the final analysis presented in this paper, we used the settings of the above procedure as unified as the natural variability of APs in different neurons allowed it.

After the initial portion of the AP in the phase-plot representation was selected according to the above formal criteria, it was fitted with (i) an exponential and (ii) a piecewise linear function.

(i) an exponential function:
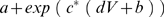



The only free parameter during the fitting procedure was the power of the exponent *c* , the remaining two parameters (*a, b*) were calculated for a given value of *c* using the linear regression in semi logarithmic coordinates. For the fitting, we used MatLab function fminbnd, which minimized the mean square deviation (MSD) of the exponential function from the data points. The minimal value of the MSD was taken as the error of the exponential fit.

Then, the same portion of the AP in the phase-plot representation was fitted with

(ii) a continuous piecewise linear function:
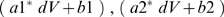



The free parameter here was the X-coordinate of the breaking point, at which two linear parts converge. Remaining parameters were calculated using the linear regression. The minimal MSD was taken as the error of linear fit.

To compare the exponential and the piecewise linear fits, we calculated the ratio of the errors:
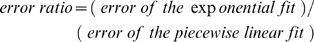



Data analysis was performed using custom-written programs in MatLab (MatLab V7.1, R14, MatWorks) environment. Data are presented as mean±SD.
