# Correction: Onset Dynamics of Action Potentials in Rat Neocortical Neurons and Identified Snail Neurons: Quantification of the Difference

**DOI:** 10.1371/annotation/947a6a14-8700-40e0-9b2d-e0d2e387d845

**Published:** 2009-07-16

**Authors:** Maxim Volgushev, Aleksey Malyshev, Pavel Balaban, Marina Chistiakova, Stanislav Volgushev, Fred Wolf

The following funding information was omitted: FW acknowledges financial support from the German Ministry for Education and Research (BMBF) via the Bernstein Center for Computational Neuroscience (BCCN) Göttingen, Germany, under Grant No. 01GQ0430. 

